# Whole-Brain Neural Dynamics of Probabilistic Reward Prediction

**DOI:** 10.1523/JNEUROSCI.2943-16.2017

**Published:** 2017-04-05

**Authors:** Dominik R. Bach, Mkael Symmonds, Gareth Barnes, Raymond J. Dolan

**Affiliations:** ^1^Wellcome Trust Centre for Neuroimaging, University College London, London WC1N 3 GB, United Kingdom,; ^2^Division of Clinical Psychiatry Research, Psychiatric Hospital, University of Zurich, 8032 Zurich, Switzerland,; ^3^Neuroscience Centre Zurich, University of Zurich, 8057 Zurich, Switzerland, and; ^4^Nuffield Department of Clinical Neurosciences, John Radcliffe Hospital, Oxford University, Oxford, OX3 9DU, United Kingdom

**Keywords:** decoding, dynamic encoding, encoding, magnetoencephalography, reward prediction

## Abstract

Predicting future reward is paramount to performing an optimal action. Although a number of brain areas are known to encode such predictions, a detailed account of how the associated representations evolve over time is lacking. Here, we address this question using human magnetoencephalography (MEG) and multivariate analyses of instantaneous activity in reconstructed sources. We overtrained participants on a simple instrumental reward learning task where geometric cues predicted a distribution of possible rewards, from which a sample was revealed 2000 ms later. We show that predicted mean reward (i.e., expected value), and predicted reward variability (i.e., economic risk), are encoded distinctly. Early on, representations of mean reward are seen in parietal and visual areas, and later in frontal regions with orbitofrontal cortex emerging last. Strikingly, an encoding of reward variability emerges simultaneously in parietal/sensory and frontal sources and later than mean reward encoding. An orbitofrontal variability encoding emerged around the same time as that seen for mean reward. Crucially, cross-prediction showed that mean reward and variability representations are distinct and also revealed that instantaneous representations become more stable over time. Across sources, the best fitting metric for variability signals was coefficient of variation (rather than SD or variance), but distinct best metrics were seen for individual brain regions. Our data demonstrate how a dynamic encoding of probabilistic reward prediction unfolds in the brain both in time and space.

**SIGNIFICANCE STATEMENT** Predicting future reward is paramount to optimal behavior. To gain insight into the underlying neural computations, we investigate how reward representations in the brain arise over time. Using magnetoencephalography, we show that a representation of predicted mean reward emerges early in parietal/sensory regions and later in frontal cortex. In contrast, predicted reward variability representations appear in most regions at the same time, and slightly later than for mean reward. For both features, representations dynamically change >1000 ms before stabilizing. The best metric for encoding variability is coefficient of variation, with heterogeneity in this encoding seen between brain areas. The results provide novel insights into the emergence of predictive reward representations.

## Introduction

Human actions are strongly determined by probabilistic predictions of future environment trajectories (Pouget et al., 2013), and in particular by estimation of future costs and benefits. Multiple brain areas represent expected reward value and its variability, the latter termed “risk” in the economic literature, and both guide action (Bach and Dolan, 2012). Expected reward value is encoded in the motor system in an action-specific manner (Guitart-Masip et al., 2011), independently from actions in the basal ganglia (Louie and Glimcher, 2012), as well as in orbitofrontal cortex (OFC) and medial prefrontal cortex (mPFC) (Louie and Glimcher, 2012; Howard et al., 2015). Additionally, reward representations are seen in early sensory cortices in both rodents (Shuler and Bear, 2006; Hui et al., 2009) and humans (Weil et al., 2010; Bunzeck et al., 2011; Doñamayor et al., 2012; Thomas et al., 2013). Expected reward variability is most prominently encoded in the OFC in nonhuman primates and humans (Preuschoff et al., 2006; Tobler et al., 2007; Mohr et al., 2010; O'Neill and Schultz, 2010; Symmonds et al., 2010). The OFC also encodes expected value, but the neural populations encoding these two features appear to be partly distinct (O'Neill and Schultz, 2010). Another area sensitive to reward variability in humans is anterior insula (Preuschoff et al., 2006; Rolls et al., 2008; Fitzgerald et al., 2010; Mohr et al., 2010; Symmonds et al., 2010), whereas a number of additional areas are reported less consistently (for review, see Bach and Dolan, 2012).

Despite progress in understanding the function of expected value and variability representations within individual areas (e.g., Walton et al., 2010; Tsuchida et al., 2010; Ogawa et al., 2013), their systems-level interplay remains elusive. This hinders insight into the neural mechanisms by which reward predictions are computed from sensory data. In particular, to constrain models that assign computational functions to individual regions, it will be necessary to elucidate how encoding in these regions evolves over time. Here, we capitalize on multivariate high-density magnetoencephalography (MEG) to investigate instantaneous neural representations at multiple time points during reward anticipation.

In our task, participants expected a probabilistic monetary gain from geometric symbols after making a correct instrumental response. Participants were overtrained on the task so as to minimize the impact of learning processes. There was no choice between options as we were not interested in decision processes. The symbols differed on two dimensions, color and fill, which predicted mean reward and reward variability. Crucially, to avoid reversal learning, perceptual features and outcome dimensions were perfectly confounded for individual participants, obviating within-subject multivariate analysis. However, by fully balancing cue-outcome associations across individuals, we could ensure these were perfectly independent across the sample, rendering possible a multivariate group analysis. In other words, we sought to assess the multivariate data patterns in the MEG signal that were shared between participants and predictive of the outcome dimension.

Importantly, multivariate models for neuroimaging data allow inference on the causal relation of independent variable and data features (Weichwald et al., 2015), by comparing encoding and decoding models (Haufe et al., 2014; Weichwald et al., 2015). Encoding and decoding models differ in the quantification of individual feature contributions. In our case, where features correspond to MEG channels with a spatial meaning, this affords a direct interpretation of channel contributions (Weichwald et al., 2015). A data channel related to an independent variable in both encoding and decoding model can be thought of being causally affected by the independent variable, which we term here “direct encoding.” If the data channel is related to an independent variable in encoding but not decoding model, it can be thought of as being “indirectly encoding” (i.e., via another data channel in the same dataset) (Weichwald et al., 2015). A data channel contributing to decoding, but not to encoding model, provides “brain state context” and must be correlated with another channel in the set but not with the independent variable (Weichwald et al., 2015).

## Materials and Methods

### 

#### 

##### Design and participants.

The study followed a 3 (expected mean reward: £1, £2, £3) × 3 (coefficient of variation: 0.08, 0.16, 0.24) factorial design. We recruited 18 healthy individuals (10 male, 8 female, 21 ± 2.28 years of age, range 18–25 years) from the general population via advertisements at University College London. All participants gave written informed consent and were fully informed about the aims of the study. The study protocol, including the form of taking written informed consent from participants, followed the principles expressed in the Declaration of Helsinki, and was approved by the National Hospital for Neurology and Neurosurgery and Institute of Neurology Joint Research Ethics Committee.

##### Independent variables and stimuli.

In a simple instrumental conditioning task, participants were trained to anticipate monetary reward signals at the offset of a visual cue presented for 2000 ms, if they made a correct action. For each cue, there were three possible positive reward outcomes (i.e., there were no losses). Visual cues were colored discs with diameter corresponding to 9.3% of the horizontal screen size. Expected mean reward was signaled by degree of fill (empty, half, full). Half fill always signaled medium reward, while the association of empty/full to low/high reward was fully balanced across participants. Reward variability was signaled by color (in RGB values from 0 to 1; blue: [0, 0, 1]; turquoise [0, 0.5, 0.5]; purple [0.5, 0, 0.5]). Color/variability association was fully balanced across participants. On each trial, participants were tasked to make two keypad responses: one to indicate fill (3 keys operated by one hand) and one to indicate color (3 keys operated by other hand). Key-fill and key-color association was fixed across participants. Because fill-mean reward and color-variability association was balanced across participants, this means that a key response was disambiguated from mean reward and variability at a group level. Association of perceptual feature (fill/variability) with left/right hand keypad was fully balanced across participants. A correct response was indicated with the word “correct!” and the actual monetary reward signal was expressed in £s. If participants failed to press 2 keys, pressed wrong keys, or pressed too late, they received a respective message and a no monetary reward signal. Feedback lasted 2000 ms during initial training and 1000 ms otherwise. During a variable intertrial interval, randomly determined to be 1000, 2000, or 3000 ms, the screen was blank. Each block was preceded by a 2000 ms, and ended with a 4000 ms, blank screen.

##### Procedure.

Participants were initially trained on a correct motor response to each cue in blocks of 12 trials. They first trained the response to the fill using black discs, and then to color using filled discs, then for both features at the same time. Each training step was repeated until participants reached 11 correct of 12 responses in a single block. In further blocks of 9 trials, we signaled “too late” if either of the two keys was pressed later than an adaptive reaction time threshold. Participants were then trained for up to 6 blocks until they showed a stable performance in responding before the threshold in 8 of 9 trials per block.

In a learning phase, participants received monetary reward signals after the visual cues in 810 trials, to achieve overtraining and minimize learning processes during MEG scanning. MEG scanning was performed either on the first or second day after training. To ensure stable performance, participants took part in refresher sessions on the MEG day and, if applicable, on the intervening day. These consisted of 180 trials during which the monetary reward signal was hidden in 50% of trials. In these trials, they just received feedback on whether their response was correct, incorrect, or too late. If they were too late on >15% over the first block of 90 trials, the reaction time threshold was increased by 50 ms for the next block of 90 trials; and if they were too late on <5%, the reaction time threshold was reduced by 50 ms. Finally, MEG scanning took place during 450 trials in a fully randomized order. Monetary reward signals were hidden in 90% of the trials to mitigate a possible impact of learning processes. The session was divided into 5 blocks of 90 trials. To motivate participants, they received the equivalent of the monetary reward signal for a random subset of 15 trials (training) or 12 trials (refreshers and MEG). Reward was determined and signaled after each of the 3 or 4 sessions (learning, 1–2 refresher, MEG) independently, and paid out after all sessions were completed.

##### Elicitation of cue utility.

After determining the reward on each session, we elicited participants' certainty equivalent for each visual cue using a Becker-DeGroot-Marschak auction (Becker and Brownson, 1964). Participants were asked to state the maximum price they were willing to expend on each visual cue. The bids were placed on a visual analog scale anchored with £0 and £4, on all 9 visual cues, in random order. For each cue, the computer would then generate a random selling price. If the computer's selling price was higher than the participant's bid, no deal was closed. Otherwise, participants would pay for their bid, and a random monetary reward signal associated with this cue is paid out on top of the pay-out from the instrumental task. From participants' certainty equivalents, c, we estimated individual risk sensitivity by fitting a standard exponential utility function with parameter a: u(c) = (1 − exp(−ca))/a.

##### MEG recordings.

MEG recordings were acquired in a magnetically shielded room (MSR) from a 275-channel CTF system with SQUID-based axial third order gradiometers (VSM MedTech Ltd), using a hardware anti-alias low pass filter of 300 Hz cutoff frequency, and sampling rate of 1200 Hz. No high pass filtering was applied. Participants made responses with an MEG-compatible response pad, held in the right hand. Visual stimuli were projected from outside the magnetically shielded room onto a screen in front of the participant. Fiducial measurements (nasion and 1 cm anterior of tragus one each side) were made using the manufacturer's procedure.

##### MEG preprocessing.

MEG data were preprocessed using standard procedures in Statistical Parametric Mapping (SPM12; Wellcome Trust Centre for Neuroimaging, London; http://www.fil.ion.ucl.ac.uk/spm). The time series was first subjected to an initial artifact correction to detect sudden jumps due to SQUID resetting, defined by a signal change between two data points exceeding 3000 fT. For these artifacts, we used a median filter >20 data points to correct the derivative of the signal, and then reconstructed the signal time series from the derivative (Bach et al., 2015). We further detected potential artifacts by searching for values exceeding 3000 fT; no such potential artifacts were found. Finally, the FieldTrip visual artifact checker was used to exclude outlier trials and channels. Trials were then epoched from 1000 ms before visual cue onset until offset. All epochs containing SQUID resettings (2.0%), visually detected artifacts (0.4%), or misdetected markers (0.1%), as well as all trials with incorrect responses by the participant (2.9%), were excluded. In 3 participants, 2 channels containing more than twice the variance of the second noisiest channel were additionally excluded. Data were then merged across blocks, low-pass filtered with a first-order Butterworth filter and cutoff frequency of 80 Hz, baseline-corrected for the time window −300 to 0 ms before cue onset, and down sampled to 200 Hz. For sensor-space analysis, individual channel data for each participant and epoch was extracted from −400 ms before cue onset to cue offset.

For source space analysis, we ensured a wide range of cortical sources were included, without biasing selection by our contrasts of interest. Thus, we reconstructed data from all trials and then selected those sources that were consistent on the group level. To this end, we used the imaging solution implemented in SPM. We used a single shell forward head model with canonical mesh (2 mm resolution), coregistered to the subject data. The model was inverted with multiple sparse priors and group inversion (Friston et al., 2008). Estimated evoked source power during visual cue presentation and across all frequencies was written out into 3D images, averaged over the 9 visual cues. These were smoothed with an 8 mm FWHM Gaussian filter, and tested across the group for consistency. We applied a lenient threshold of *p* < 0.05 uncorrected and identified 25 activity peaks. We then extracted the reconstructed source activity from spheres with 5 mm radius around each of these peaks, and retained the eigenvariate time course for that location. Thirteen of these time courses contained pairwise independent signals and were retained; each set of mutually collinear sources is shown in figures as combined source. All soures are visualized for better recognizability with larger spheres in the figures of this paper (10 mm radius for superficial sources, 15 mm for a deep cuneus source, and 20 mm for a cingulum source).

##### MEG analysis.

Sensor and source data were analyzed using the same algorithm (see Fig. 2). Because there were more trials than sensors or sources, we could use standard multivariate methods, namely, MANOVA. For each participant, perceptual features (fill, color) and predicted outcome dimensions (mean reward, variance) are perfectly confounded, such that patterns related to physical features or to reward attributes cannot be disambiguated at a single-subject level. However, they were perfectly orthogonal at the group level; consequently, all data analysis was performed at the group level. While the explained variance in the MEG signal across the group is necessarily low due to large between-subjects variability in the MEG patterns, the combination of data points from participants lends this analysis high statistical power.

We created time bins of 10 ms duration (corresponding to 2 time points after down sampling) and averaged data within the time bins, resulting in 240 (sensor level: −400 to 2000 ms wrt cue onset) or 200 (source level: 0–2000 ms wrt cue onset) bins. For each time bin, we concatenated all trials from all subjects into a data matrix *X* that contains as many columns as there are data channels (see Fig. 2). Statistical analysis was done in R (www.r-project.org) using the functions manova for the encoding, and lm for the decoding model. Results from this analysis were corrected for the number of time bins by using a cluster-level permutation test (Maris and Oostenveld, 2007). Subsequent tests for individual channel contributions in the encoding model were done using the functions lm and anova.

Specifically, encoding of stimulus feature *Y* (mean reward, variability, fill, color) and data *X* was tested in a MANOVA as follows:

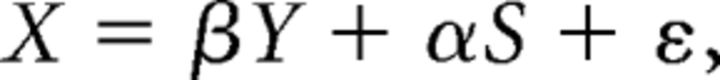
 where *X* is a data matrix with 275 (sensor level) or 13 (source level) columns, *S* encodes a subject specific intercept term, and the statistical test is done on the *F*-transformed Pillai–Bartlett trace. In time bins with significant encoding after cluster-level correction, we then tested the contribution of each individual data channel *X_k_* (sensors/sources) in regression models of the following form:

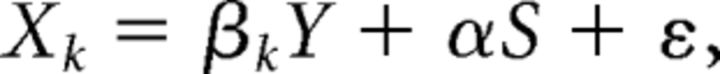
 without correction.

Decoding of stimulus features *Y* from data *X* was tested in an ANOVA as follows:

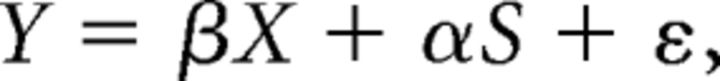
 with a standard *F* test. For time bins significant after cluster-level correction, we tested the contribution of each individual channel *X_k_* by leaving it out of the model, and comparing the reduced with the full model by an *F* test.

One crucial difference between these two approaches lies in the quantification of individual channel contributions, and thus in the interpretation of these contributions (Haufe et al., 2014; Weichwald et al., 2015). The single-channel encoding model detects shared variance between stimulus feature and data channel. It will thus detect any relation between stimulus feature and data channel, independent of the other data channels. By contrast, in the decoding model, all channels are used to predict the stimulus feature, and the individual channels are tested by leaving each channel out of the model. A channel encoding the stimulus feature via another data channel (“indirect encoding”) will not improve the prediction of the stimulus feature and thus not show up in the decoding model as significant. At the same time, a channel unrelated to the stimulus feature but related another channel may remove noise variance from these data channel and thus improve the decoding. Such channels may be referred to as “brain state context” (Weichwald et al., 2015) because they relate to an encoding channel but do not themselves encode.

We only analyzed individual data channels at those time bins where the multivariate model was significant after correction for multiple comparison. The decision algorithm for the causal contribution was based on Weichwald et al. (2015) and implemented as follows:

1. Channel contribution significant (*p* < 0.05) in the encoding model
  - Channel contribution significant (*p* < 0.05) in decoding model: direct cause
  - Channel contribution nonsignificant (0.5 < *p* < 0.10) in the decoding model: possible direct cause
  - Channel contribution nonsignificant (*p* > 0.10) in the decoding model: indirect cause
2. Channel contribution significant (*p* < 0.05) in the decoding model
  - Channel contribution significant (*p* < 0.05) in the encoding model: direct cause
  - Channel contribution nonsignificant (0.5 < *p* < 0.10) in the encoding model: possible direct cause
  - Channel contribution nonsignificant (*p* > 0.10) in the encoding model: brain state context

In this approach, a nonsignificant effect in one model is only interpreted if there is a significant effect in the other model. Cases in which both models were nonsignificant (0.5 < *p* < 0.10 or *p* > 0.10) were not considered.

Temporal stability of representations was assessed on source data using a cross-prediction approach. We applied each encoding MANOVA model, fitted at a source time bin, to target data from each of the other time bins, and scored explained variance in the target data. Within each target time bin, we randomly permuted trial labels 1000 times to establish the distribution of explained variance in the target data under the null hypothesis that the source model is not predictive of the target data. Cross-prediction data points with *p* > 0.05 or with negative explained variance were set to zero for visualization and further analysis. The pattern in the cross-prediction matrices was quantified by summing all off-diagonal cross-prediction values relating to data and model from the first half of the anticipation window (0–1000 ms) and subtracting this from the sum of the second half (1000–2000 ms). We then randomly permuted time labels of the cross-prediction matrix 100,000 times and recomputed the sums, to establish the empirical distribution under the null hypothesis that cross-prediction performance is uniform across time, and thus statistically test the pattern in the cross-prediction matrices. The same test was done on the diagonal of the cross-prediction matrix, to score the encoding strength in the original encoding model. To verify that cross-prediction performance was not driven by differences in the model fit at the source time bin, we divided cross-prediction values in all target time bins by the explained variance of the fitted model at the source time bin, and repeated the analysis of the cross-prediction matrix.

Finally, we tested which variability metric best explains our data, by using Bayesian model comparison of encoding models with different metrics. Residual sums of squares from these models were converted to Akaike Information Criterion (AIC) (Raftery, 1995). SD and variance are correlated with mean reward in our design, and these metrics were therefore decorrelated by subtracting the mean of that metric within each mean reward level.

To render more plausible that our results in relation to overall mean/variability encoding were not driven by one or a few individual participants, we split the dataset into three subgroups balanced for cue/outcome association and reanalyzed the overall time course of encoding/decoding across sources in these three subgroups. This analysis provided no evidence that our results were driven by individual subjects.

To validate our approach, we confirmed that perceptual stimulus features were encoded/decodable in/from our data. After correction for multiple comparison (*p* < 0.05 cluster level), both encoding and decoding model were first significant on the sensor level 70 ms (fill) and 80 ms (color) after cue onset, and throughout the remaining 193/192 time bins of the cue presentation period, with the exception of 7 time bins (fill) or 12/29 time bins (encoding/decoding for color), but not in the 40 time bins before cue onset. On the source level, encoding/decoding models for fill were first significant after 110 ms and throughout the remaining 189 time bins of the cue presentation period with the exception of 3/8 time bins (encoding/decoding). For color, both models were first significant after 130 ms and for 88/69 (encoding/decoding) of the remaining 188 time bins in the cue presentation period.

## Results

**Figure 1.**
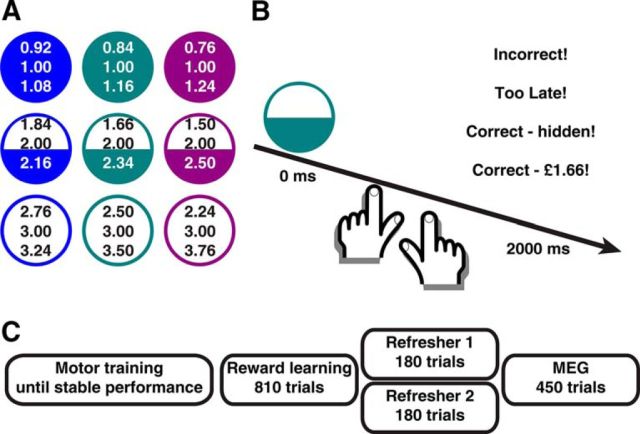
Experimental procedure. ***A***, Visual cues with the three possible outcomes (i.e., there were no losses). Fill of circles represents mean reward. Color represents variability. Cue-outcome and response-outcome mapping was fully balanced across participants. ***B***, Intratrial procedure. A reward predictor was shown for 2000 ms, during which participants indicated color and fill with one button press per feature. At offset, one of four possible messages appeared. ***C***, Procedure. Participants were overtrained beforehand. A total of 90% of outcomes were hidden during MEG, to suppress a possible impact of ongoing learning processes.

**Figure 2.**
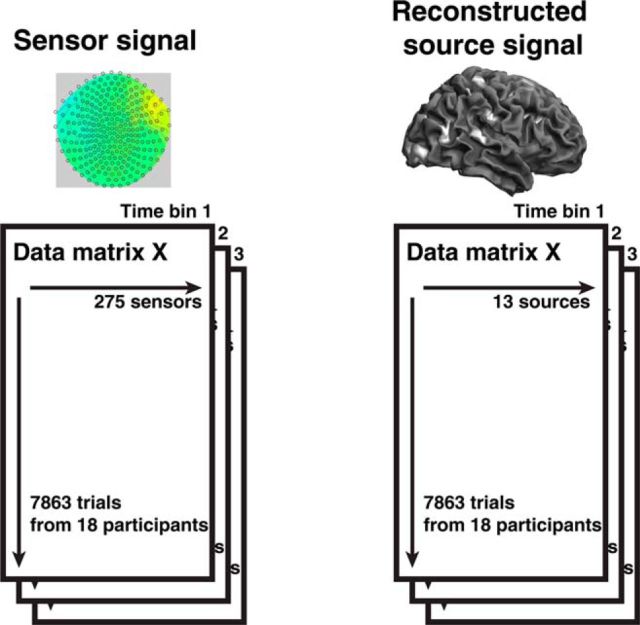
Analysis scheme. Sensor data and reconstructed source data were averaged within 10 ms time bins and concatenated across participants. The data matrix *X*, together with the trial-by-trial stimulus variable of interest *Y* (mean reward, variability, or perceptual feature), was then fed into multivariate analysis.

Participants were overtrained to press a unique combination of 2 keys following presentation of each of 9 cues, within a response time window adapted to ensure 85%–95% correct performance. Each cue predicted the occurrence of a reward after 2000 ms if the correct keys were pressed. The reward had one of 3 mean and variability (coefficient of variation) levels, respectively (Fig. 1). We recorded MEG in a session in which outcomes were largely omitted to reduce any impact arising out of ongoing learning processes. We analyzed sensor data and then reconstructed sources where overall activity showed the highest consistency across the group (Fig. 2). To validate our approach, we ensured that perceptual features, which at a group level were orthogonal to reward features, could be decoded from the data during the anticipation window.

### Sensor level

**Figure 3.**
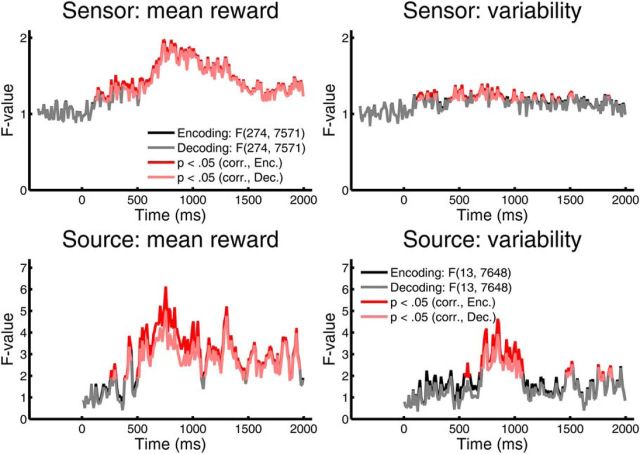
Predicted reward representation in sensor and source signal patterns across participants at different time points. Encoding (black) and decoding (gray) of predicted mean reward and variability from MEG sensor signals and from reconstructed source activity. *F* values refer to *F*-transformed Pillai–Bartlett trace from the encoding MANOVA, or to *F* ratio of a decoding ANOVA, and are computed using pooled error variance, as reflected in degrees of freedom. Red (encoding) and pink (decoding) lines indicate significant time bins (*p* < 0.05) after a cluster-level based permutation test to account for multiple comparison across time.

Mean reward was first encoded in MEG sensor data 110 ms after cue onset, and first decoded after 120 ms (Fig. 3). Encoding/decoding were seen throughout the remaining cue presentation period. Reward variability was first encoded/decoded after 110 ms. We then established the contribution of individual sensors to reward encoding, by mapping the time points of initial and maximal contribution for every sensor. We could not detect any particular spatial configuration in this analysis. As each sensor contains signals from various different sources, we projected the sensor data onto 13 reconstructed sets of sources to enable a more precise spatial specification (Table 1).

**Table 1.** Reconstructed sources considered for analysis*^a^*

Source location (number in Fig. 4)	MNI peak coordinates (mm)	First significant				Preferential encoding (Mag/Var)	Best fitting variability metric
		Mean reward (ms)		Variability (ms)			
		Encoding	Decoding	Encoding	Decoding		
Occipital							
L calcarine (1)	−2/−96/6	295	285	1005	885	Mag	CV
	−2/−94/−4						
	−2/−88/14						
R/L cuneus, L precuneus (2)	16/−70/28	295	295	745	725	Var	SD
	−16/70/30						
	−14/−76/24						
R inferior occipital (3)	40/−88/−8	385	425	545	715	Mag	CV
	30/−96/−8						
Temporal							
R/L STG (4)	−62/−32/10	255	395	575	735	Var	CV
	58/−34/8						
R/L MTG (5)	52/−62/14	255	735	845	805	Mag	V
	−54/−62/8						
	−42/−56/10						
Parietal							
R supramarginal (6)	54/−24/30	255	425	545	725	Var	SD
R postcentral (7)	40/−36/60	645	385	—	1805	Mag	V
	34/−34/54						
L postcentral (8)	−56/−20/26	255	435	545	825	Var	SD
L postcentral (9)	−40/−38/60	515	385	745	925	Mag	V
Frontal							
R/L mid cingulum (10)	−6/12/38	505	715	1005	775	Mag	CV
	8/6/44						
L medial orbital (11)	−10/62/−4	535	625	745	845	Mag	CV
	−6/62/−12						
L precentral (12)	−36/4/32	585	385	545	705	Var	SD
	−40/2/42						
R IFG triangular part (13)	32/18/24	305	555	545	1475	Mag	SD

*^a^*Timing is given as center of analysis time bins. Preferential encoding: lowest AIC summed across all time bins (absolute difference >3) for encoding models with mean reward or variability as predictor (choice of variability metric did not impact this result). Best fitting variability metric: lowest AIC summed across all time bins (absolute difference >3) for encoding models with CV, SD, or variance (V) as predictor.

### Source level

**Figure 4.**
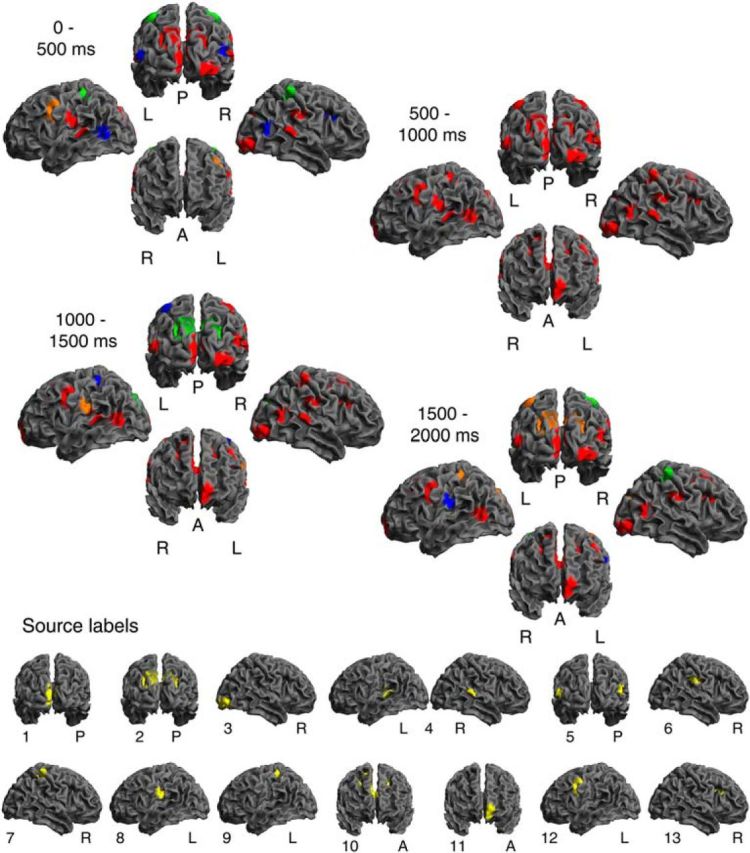
Representation of mean reward. Results are summarized over 500 ms intervals. Individual time bins are shown in Movie 1 and Figure 6. Description of the source labels in Table 1. Red represents direct encoding (i.e., individual source significant in encoding model in at least one overall significant time bin in this interval, and also significant in decoding model in at least one time bin). Orange represents likely direct encoding (significant in either encoding or decoding, and undecided in the other). Blue represents indirect encoding (no decoding, but significant in encoding model). Green represents nonencoding, but correlated with encoding regions (no encoding, but significant decoding).

**Figure 5.**
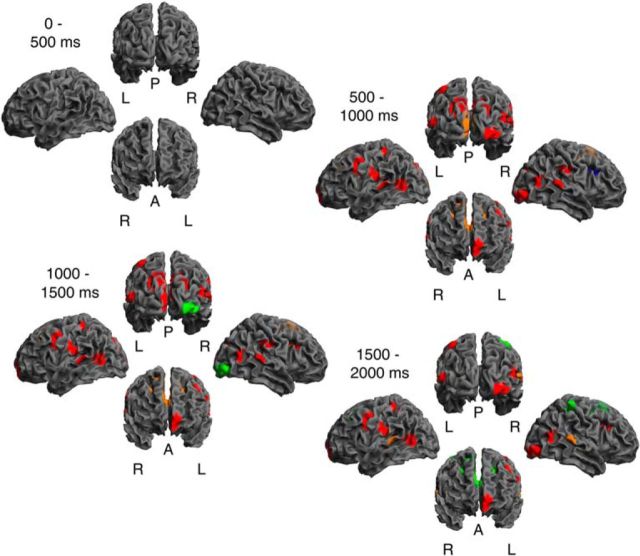
Representation of reward variability. Results are summarized over 500 ms intervals. Individual time bins are shown in Movie 2 and Figure 6. Description of the source labels in Table 1. Red represents direct encoding (i.e., individual source significant in encoding model in in at least one overall significant time bin in this interval, and also significant in decoding model in at least one time bin). Orange represents likely direct encoding (significant in either encoding or decoding, and undecided in the other). Blue represents indirect encoding (no decoding, but significant in encoding model). Green represents nonencoding, but correlated with encoding regions (no encoding, but significant decoding).

**Figure 6.**
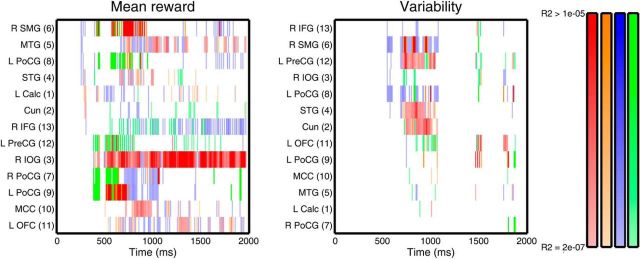
Representation of reward mean and variability. For each source, representation in each significant time bin is color-coded. Saturation reflects explained variance. Red represents direct encoding (i.e., individual source significant in encoding model and decoding model). Orange represents likely direct encoding (significant in either encoding or decoding, and undecided in the other). Blue represents indirect encoding (no decoding, but significant in encoding model). Green represents nonencoding, but correlated with encoding regions (no encoding, but significant decoding). Description of the source labels in Table 1.

At the source level, mean reward was first encoded in the MEG signal 250 ms after cue onset and first decoded after 280 ms. Reward variability was first encoded 540 ms and decoded 700 ms after cue onset. The fact that sensor level models showed an earlier emergence of the reward encoding indicates some loss of information through the source projection. However, the source projection, with its greater spatial precision, enabled us to analyze the contribution of individual sources in greater detail, where we observed a much more pronounced spatial pattern than at a sensor level (Figs. 4–6; Movies 1, 2; Table 1). In this analysis, we capitalized on a differential interpretation of source contributions to encoding and decoding models (see Materials and Methods), thus separating between sources directly or indirectly encoding stimulus feature, and those providing brain state context (i.e., improving the decoding) but by themselves not encoding the feature.

Movie 1. Representation of mean reward, for individual 10 ms time bins. Description of the source labels in Table 1. Red represents direct encoding (i.e., individual source significant in encoding model in at least one overall significant time bin in this interval, and also significant in decoding model in at least one time bin). Orange represents likely direct encoding (significant in either encoding or decoding, and undecided in the other). Blue represents indirect encoding (no decoding, but significant in encoding model). Green represents nonencoding, but correlated with encoding regions (no encoding, but significant decoding). 10.1523/JNEUROSCI.2943-16.2017.video.1

Movie 2. Representation of reward variability, for individual 10 ms time bins. Description of the source labels in Table 1. Red represents direct encoding (i.e., individual source significant in encoding model in at least one overall significant time bin in this interval, and also significant in decoding model in at least one time bin). Orange represents likely direct encoding (significant in either encoding or decoding, and undecided in the other). Blue represents indirect encoding (no decoding, but significant in encoding model). Green represents nonencoding, but correlated with encoding regions (no encoding, but significant decoding). 10.1523/JNEUROSCI.2943-16.2017.video.2

Mean reward was first encoded in temporoparietal and occipital sources, followed by frontal sources, with OFC emerging as the last source after >500 ms (Figs. 4, 6; Movie 1). Interestingly, a source in visual cortex (right inferior occipital gyrus, source 3) showed the most consistent direct encoding across the entire cue presentation period. Several sources encoded brain state context, but not mean reward, over a large portion of cue presentation time. In other words, these sources were correlated with other sources encoding mean reward and thus contributed to the decoding performance but did not encode mean reward itself. The first emerging sources were labeled as indirectly encoding during early time bins. Because we did not identify any directly encoding source at these time bins, this may indicate that all sources encoded mean reward in a relatively similar way during these time bins such that their omission from the decoding model did not reduce decoding performance.

In contrast, reward variability was encoded in a more sparse set of sources, which again included visual areas. These sources became active after ∼500 ms, ceased their activity after ∼1500 ms, and several did so within similar time bins (Figs. 5, 6; Movie 2). Thus, there was a less pronounced spatial unfolding over time. A source in visual cortex (Cuneus, source 2) was labeled as directly encoding variability; this source was distinct from the one directly encoding mean reward.

To directly compare the encoding of mean reward and variability, we quantified evidence of the encoding models in each time bin as AIC. Mean reward explained significantly more variance in the data (i.e., had lower AIC values with a difference > 3) than variability across time bins. Sources preferentially encoding mean reward or variability are listed in Table 1. Crucially, in all four lobes, there was at least one source preferentially encoding mean reward and one source preferentially encoding variability.

### Temporal stability of reward representations

Up to now, we have considered spatial, but not temporal, stability of representations. That is, even if the same spatially stable set of regions encode reward statistics at different time points, these representations may still be distinct for each time bin within the set of sources, and thus temporally unstable. Hence, we next addressed temporal stability of predicted reward representations, by applying the encoding model fitted at one time point, to the data from another time point, and measuring explained variance. Significance of these cross-predictions was established by randomly permuting trial labels.

**Figure 7.**
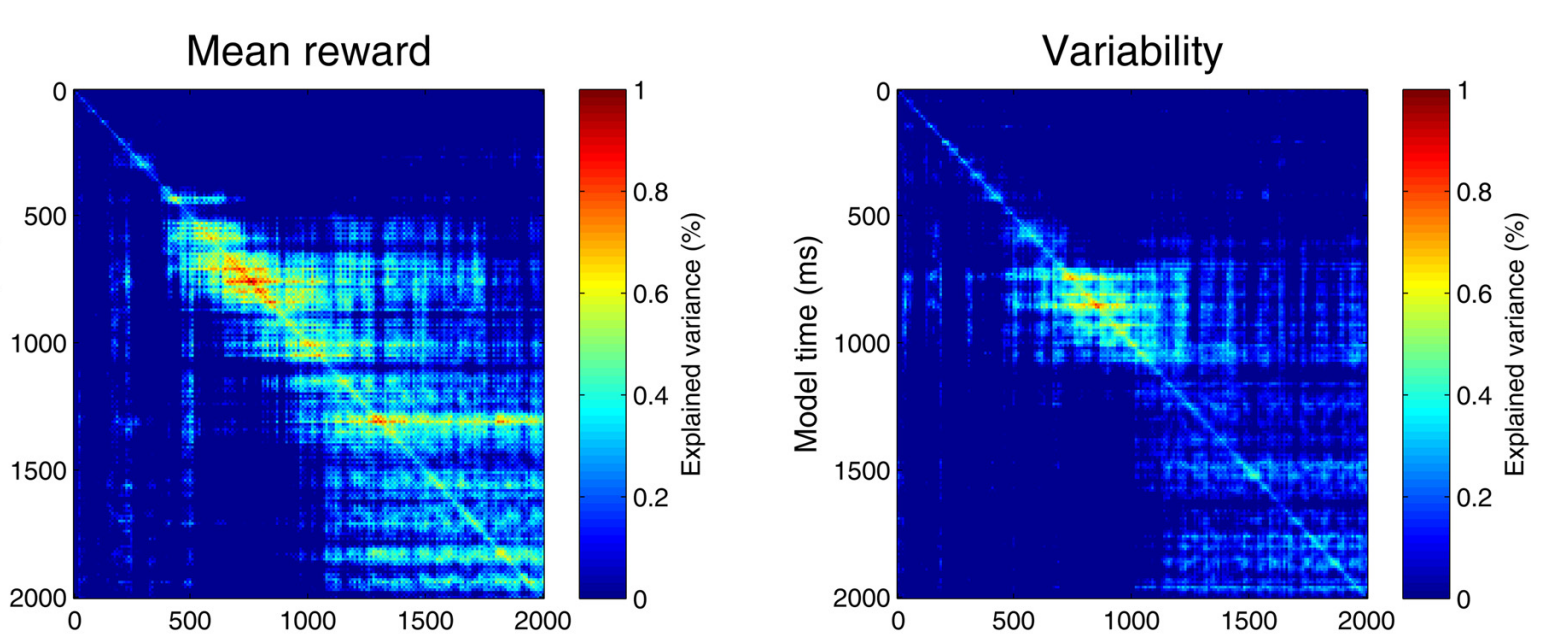
Temporal stability of reward representation. Cross-prediction matrix for the prediction of data points from encoding MANOVAs fitted at other data points. Nonsignificant cross-prediction (random permutation test), and negative explained variance, is set to zero (dark blue). Temporal stability is higher in the second than in the first half of the anticipation window (random permutation of cross-prediction matrix, *p* < 0.001), although encoding is equal (mean) or stronger in the first half (variability).

Results are shown in Figure 7, where all time points with nonsignificant cross-prediction, or negative explained variance, are set to zero. The main diagonal of the figure shows how well reward features are encoded at each point in time. Off-diagonal values show how similar the encoding is at other time points. Although encoding of mean and variability peaked at ∼800–1000 ms (see also Fig. 3), the stability of the representation (i.e., how well the data could be predicted from models fitted at other time bins) increased in the second 1000 ms. Random permutation of the cross-prediction matrix demonstrated a significantly more stable encoding in the second than in the first half of the anticipation window both for mean reward (*p* < 0.0001) and for reward variability (*p* = 0.0002). The same results were found when accounting for explained variance in the initial model fit. In contrast, in the initial model, we found no overall difference between first and second half for mean reward, and variability encoding was stronger in the first than in the second half (*p* < 0.0001). In sum, stronger cross-prediction in the second half of the anticipation window reflects increased temporal stability of representations and not a stronger encoding in the time bins in which the model was initially fitted.

### Variability metric

Hitherto, we quantified reward variability as coefficient of variation (CV), which is orthogonal to mean reward. Next, we addressed whether the identified brain areas actually use CV as a metric to encode variability, or whether MEG source signals are better explained by SD or variance (V) of reward. Because SD and V are not independent of mean reward, these variables were decorrelated from mean reward for this analysis, by subtracting the average value of SD or V within each mean reward level. Combining evidence relating to all data from all sources, CV emerged as a clear winner (AIC difference > 120). Nevertheless, we observed that, at the sensor level, decorrelated SD explained the data better than CV (AIC difference > 2400) or V (AIC difference > 1700).

To explain the above discrepancy, we note that individual sources map onto a different number of sensors, depending on their location. Hence, if different sources encode different metrics, this could explain the anisotropy between source and sensor level. Indeed, heterogeneity was observed in individual sources, most of which were best explained by CV, but some by SD or V (Table 1). Together, these results suggest that different regions may encode reward variability using different metrics.

Finally, we used a cross-prediction approach to rule out a possibility that our variability encoding results are driven by nonlinear utility functions. According to expected utility theory (von Neumann and Morgenstern, 1944), increasing mean reward relates to higher expected utility, but increasing reward variability relates to lower expected utility if an agent is risk-averse (as is the case for most human subjects, e.g., Levy et al., 2010). Although our behavioral results did not indicate homogeneous utility functions across the group, it is still a concern that variability encoding is really a negative utility encoding and in this case would be very similar to negative mean reward (value) encoding. Here, we find that encoding models for mean reward did not explain data relating to variability or negative variability, at any point in time.

### Certainty equivalents

Finally, we analyzed to what extent participants' valuation of the cues impacted their explicit preferences. Certainty equivalents depended on signaled mean reward (*F*_(1,315)_ = 296.29, *p* < 1e-47) but not on signaled variability (*F*_(1,315)_ < 1, *p* > 0.50). They were higher after MEG than after initial training (*F*_(1,315)_ = 16.96, *p* < 0.0001). Mean ± SE of certainty equivalents for the three mean reward levels (averaged across sessions) were 0.92 ± 0.12, 1.65 ± 0.16, and 2.58 ± 0.22, and for training and MEG (averaged across mean reward levels), 1.56 ± 0.23 and 1.88 ± 0.22, respectively. The mean certainty equivalents elicited by the BDM procedure were lower than the true expected value of the cues, which reflects sensitivity to reward variability (economic risk). An exponential utility function fit the certainty equivalents better (AIC difference > 50) than the true expected values. Estimated parameters of this function indicated an average (SD) risk sensitivity of a = 0.13 (0.26) after initial training, and *a* = 0.05 (0.15) after MEG. Overall, participants were neither significantly risk averse nor risk seeking (after initial training, *t*_(1,15)_ = 2.03, *p* = 0.06; after MEG, *t*_(1,17)_ = 1.38, *p* = 0.19): there was considerable population heterogeneity with (initial training/MEG) 11/10 subjects showing risk aversion, and 5/8 showing risk seeking. Finally, there was no impact of either mean reward or reward variability on reaction times or accuracy of responses, possibly due to the adaptive reaction time thresholds, and high level of overtraining.

## Discussion

Investigating the spatiotemporal dynamics of predicted mean reward and variability using human high-density, multivariate MEG revealed four main findings. First, mean reward and variability of the reward distribution are encoded in MEG sensor signals after ∼110–120 ms until the end of a predictive cue presentation. Second, both reward statistics are encoded in reconstructed source activity with the representation of mean reward emerging over time and first seen in polymodal and visual areas, and later in prefrontal cortex with a protracted emergence within OFC. On the other hand, variability encoding was observed in a similar set of sources, including early visual areas, but arose almost simultaneously across many sources. Third, the temporal stability of source encoding increased over time. During the second 1000 ms of the anticipation window, source activity was to some extent explained by encoding models fitted at a different time point, and this was less pronounced in the first 1000 ms. Finally, as a variability metric, CV best explained the set of reconstructed source activity and also most individual sources. Some sources were, however, better explained by SD or variance, and this was also the case at the sensor level, which may indicate some heterogeneity across brain areas.

Previous studies have identified encoding of predicted mean reward in frontal and sensory areas (Bunzeck et al., 2011; Doñamayor et al., 2012; Louie and Glimcher, 2012; Howard et al., 2015). By elucidating the temporal unfolding of such representations over time, and in particular an increase in temporal stability, our current data complement these findings. According to our results, mean reward computation is first seen in parietal and visual areas. Within our set of sources, the spatiotemporal pattern of mean reward encoding seems to endorse a bottom-up process from occipital/parietal to frontal regions. Whether there are top-down influences from sources that we were unable to detect with MEG remains a question that we cannot finally disambiguate.

The representation of predicted reward variability is a more controversial issue where there is a reported inconsistency in human neuroimaging studies (Bach and Dolan, 2012). We found a similar set of brain regions as that seen for mean reward encoding, but this encoding arises later and shows a distinct spatiotemporal profile. A novel finding in this context is a reward variability encoding in early sensory areas, something that parallels the mean reward representations observed in previous studies (Shuler and Bear, 2006; Hui et al., 2009; Weil et al., 2010; Bunzeck et al., 2011; Doñamayor et al., 2012; Thomas et al., 2013). Indeed, in our study, a source in the cuneus preferentially encoded reward variability rather than mean reward.

Although the temporal profile of mean reward encoding suggests a hierarchical processing stream from sensory/polymodal to frontal areas, this pattern is less pronounced for variability encoding. Nevertheless, the OFC emerged later than parietal and visual sources. As for mean reward encoding, variability encoding appears to emerge over 1000 ms and stabilized in the second half of the anticipation interval. Interestingly, behavioral studies in humans suggest that the computation of higher statistical moments in predicted reward distributions may take more time than to compute the mean (Nursimulu and Bossaerts, 2014), which could explain a later emergence of variability encoding. It is a possibility that overtraining reduces this computation time and thus the amount of training may influence the precise time course of reward feature representations.

Interestingly, CV was the best-fitting metric to explain variability encoding across all MEG sources. Unlike SD or variance, CV is scaled by expected mean reward. This observation bears on previous findings that nonhuman animals' (birds' and insects') as well as human choices are better predicted from CV than variance, for humans in particular when decision statistics are learned from experience rather than presented propositionally (Weber et al., 2004). In our task, reward distributions were learned. Because learned and propositional reward distributions are suggested to have a somewhat distinct neural encoding (Fitzgerald et al., 2010), our finding that CV best explains variability representations may be restricted to learned distributions. Interestingly, however, we found a heterogeneity across brain regions with respect to the best encoding metric (i.e., the signal in some sources was better explained by SD or variance rather than CV).

Primate electrophysiological studies highlight a role for the orbitofrontal cortex in the acquisition and maintenance of variability encoding (O'Neill and Schultz, 2010), a finding replicated in the current dataset. On the other hand, human fMRI studies have reported involvement of diverse sets of brain areas. These regions encompass the OFC (Preuschoff et al., 2006; Tobler et al., 2007; Mohr et al., 2010; Symmonds et al., 2010), ACC (Preuschoff et al., 2006; Christopoulos et al., 2009), anterior insula (Preuschoff et al., 2006; Rolls et al., 2008; Fitzgerald et al., 2010; Mohr et al., 2010; Symmonds et al., 2010), temporal (Preuschoff et al., 2006) and parietal cortex (Preuschoff et al., 2006; Symmonds et al., 2011), basal ganglia (Dreher et al., 2006; Preuschoff et al., 2006; Symmonds et al., 2010), and midbrain (Aron et al., 2004; Preuschoff et al., 2006), where only a subset of these regions is reported in each individual study. The aforementioned studies were heterogeneous in terms of the behavioral tasks used, in relation to whether reward distributions were learned or explicitly signaled, and in the number of learning trials. Incomplete learning of reward distributions entails uncertainty about these distributions, termed ambiguity in the economic literature (Ellsberg, 1961) and associated with specific neural representations (Huettel et al., 2006; Bach et al., 2009, 2011). We circumvented some of these issues by overtraining participants in a simple instrumental task without choice, and by not revealing most outcomes during MEG scanning so as to suppress ongoing learning. Finally, some neuroeconomic models based on expected utility theory posit that reward variability should only be encoded via its impact on subjective utility (von Neumann and Morgenstern, 1944; Camerer, 1995). This means that the effect of increasing mean reward or decreasing reward variability should be identical. Crucially, and extending a previous primate report on variability encoding in OFC (O'Neill and Schultz, 2010), we can rule out by cross-prediction a possibility that our results in relation to variability encoding are driven by a negative utility encoding.

Although we highlight a rich and distributed network of reward representations, the precise function of different brain regions remains to be determined. For example, the OFC appears critical for learning from reward feedback (Tsuchida et al., 2010; Walton et al., 2010) and thus would seem to use reward statistics. Other regions that we show tracking reward statistics may not necessarily use them to compute decisions, as not all neural population encoding decision variables impact on a decision (Katz et al., 2016). Finally, variability encoding in the OFC (O'Neill and Schultz, 2010) has been suggested to reflect an encoding of salience because cues predicting more uncertain outcomes are also more salient (Ogawa et al., 2013).

A previous MEG experiment found reward variability encoding only in reconstructed source activity (Symmonds et al., 2011), whereas here we demonstrate variability encoding in sensor-level signals across the anticipation interval, lending greater credence to the robustness of our findings. Furthermore, we validate our approach by showing that physical features of the stimuli could be as well reconstructed from the data as reward features. Finally, our study was not designed to address a question whether participants were behaviorally sensitive or insensitive to the level of reward variability.

In conclusion, we show that visual and parietal areas encode predicted reward variability as well as mean reward, and demonstrate a temporal unfolding of such representations over time. A specification of the temporal dynamics of decision making can add richness to an understanding of how regions compute predictions as well as communicate with each other, thereby enabling optimal action selection.
